# The Detection of a Functional 168 bp Deletion of the *HOXB13* Gene Determining Short Tail and Its Association with Senior Growth Traits in Sheep Breeds Worldwide

**DOI:** 10.3390/ani14111617

**Published:** 2024-05-29

**Authors:** Qihui Zhu, Peiyao Liu, Mingshi Zhang, Yuxin Kang, Linmi Lv, Hongwei Xu, Qingfeng Zhang, Ran Li, Chuanying Pan, Xianyong Lan

**Affiliations:** 1 College of Animal Science and Technology, Northwest A&F University, Yangling 712100, China; zqhzqhzqh2002@163.com (Q.Z.); liu_peiyao@126.com (P.L.); kangyuxin990610@163.com (Y.K.); 18337557860@163.com (L.L.); ran.li1986@hotmail.com (R.L.); panyu1980@126.com (C.P.); 2 College of Veterinary Medicine, Northwest A&F University, Yangling 712100, China; zhangmingshi2021@163.com; 3 College of Life Science and Engineering, Northwest Minzu University, Lanzhou 730030, China; xuhongwei@xbmu.edu.cn; 4 Tianjin Aoqun Sheep Industry Academy Company, Tianjin 300000, China; zhangqf@aoqunmuye.com

**Keywords:** sheep, *HOXB13* gene, insertion/deletion (InDel), growth traits

## Abstract

**Simple Summary:**

Simple Summary: Sheep breeding has important economic value in China’s animal husbandry industry, and growth traits represent an important factor affecting the economic benefits of the industry. This study is based on a functional 168 bp SINE element insertion upstream of the *HOXB13* gene discovered in GWAS. By using whole-genome sequencing (WGS) data, we analyzed the frequency of the *HOXB13* gene in 33 different sheep breeds worldwide, and we genotyped 6 specific sheep breeds by using PCR and agarose gel electrophoresis. We also associated the polymorphism of the *HOXB13* gene with the growth traits of Luxi black-headed sheep and found that the 168 bp insertion showed a certain correlation with the growth traits in sheep. This may provide effective assistance for improving the economic benefits of the sheep industry.

**Abstract:**

In recent years, genome-wide association studies (GWAS) have uncovered that the *HOXB13* gene is a key regulatory factor for the tail length trait of sheep. Further research has found that there is a functional 168 bp SINE element insertion upstream of the *HOXB13* gene, which leads to the occurrence of long tails in sheep. However, the frequency of mutations in the 168 bp SINE element of the *HOXB13* gene among different sheep breeds around the world and its relationship with growth traits are still unclear. This study used whole-genome sequencing (WGS) data, including 588 samples from 33 different sheep breeds around the world, to evaluate the frequency of *HOXB13* gene mutations in different sheep breeds globally. At the same time, this study also selected 3392 sheep samples from six breeds. The genetic variation in the 168 bp InDel locus in the *HOXB13* gene was determined through genotyping, and its association with the growth traits of Luxi black-headed sheep was analyzed. The research results indicate that the polymorphism of the 168 bp InDel locus is significantly correlated with the hip width of adult ewes in the Luxi black-headed sheep breed (*p* < 0.05) and that the hip width of adult ewes with the DD genotype is significantly larger than that of adult ewes with the ID genotype (*p* < 0.05). This study indicates that there is consistency between the research results on the sheep tail length trait and growth traits, which may contribute to the promotion of sheep breed improvement.

## 1. Introduction

In recent years, the demand for livestock products in the market has significantly increased. Sheep hold important economic value in the animal husbandry industry and can provide people with a series of animal by-products [1]. Tail length is one of the most important phenotypes of sheep and is of great significance for the development of animal breeding and animal husbandry. At present, most modern sheep breeds are long-tailed, but long-tailed sheep have a series of defects, such as being more susceptible to diseases, issues with natural mating and the reproductive rate, and the emergence of wool pollution and decreased wool quality [2]. Growth traits are among the most important economic traits of sheep and represent a significant factor affecting the economic benefits of the industry. At present, the growth traits of sheep still need to be improved. Previous research suggests that there may be a latent connection between tail length and growth traits in sheep [3,4]. However, this potential relationship remains unclear and warrants further investigation to elucidate it. Owing to various limitations, there are challenges in sheep breeding, and the economic benefits of the sheep industry require refinement [5,6,7]. Therefore, strengthening breeding and improving tail length traits and growth traits by selecting sheep breeds with excellent traits can effectively promote the development of the industry.

Genome-wide association studies (GWAS) have been increasingly used to identify genes related to phenotypes. They involve an association analysis between detected phenotype data and genotype information obtained from population sequencing, accurately locating SNP loci that may be related to phenotype data, and mining genes related to phenotypes [8]. Recently, studies have incorporated structural variation (SV) into GWAS on plants, accurately locating causal structural variations in various agronomic traits of crops such as rice, corn, and cotton [9,10,11]. At the same time, marker-assisted selection (MAS) has been widely used in animal breeding [12,13,14,15]. It offers advantages such as convenient detection, low time consumption, low cost, accuracy, strong operability, and no interference from environmental conditions. It can greatly shorten the breeding period, significantly improve the accuracy of selection, and greatly enhance breeding efficiency. Therefore, it is increasingly being utilized in breeding work.

*Hox* genes are considered developmental genes that regulate the activity of specific cells throughout an organism’s lifetime [16]. During embryogenesis, the combinatorial pattern of *Hox* gene expression along the anterior–posterior (head-to-tail) and proximal–distal (center-to-periphery) axes is relatively strictly defined, and this positional information is maintained into adulthood [17]. In animals, *Hox* genes coordinate and control multiple growth and development systems, influencing the development and differentiation of limbs, the brain, viscera, muscles, blood, and skeletal structures [18]. Previously, our team discovered the *HOXB13* gene through genome-wide association studies (GWAS), which is a key regulatory factor for sheep tail length and has a significant impact on this trait [19]. *HOXB13* is the gene with the highest expression at the 5′ end in the *HoxB* cluster, expressed in the posterior region of developing embryos. The characteristic expression pattern persists until day 12.5 and is confined to the tail of the spinal cord and tail bud, as well as the extent of the urogenital sinus [20]. The expression of *HOXB13* closely corresponds with the dynamic changes associated with the formation of secondary neural tubes (SNTs) and tail development [21]. In order to determine the role of the *HOXB13* gene in tail development, several studies have generated alleles with a functional deficiency in *HOXB13* through gene targeting. The viability and fertility of heterozygous and homozygous mutants are normal; however, the tails of homozygous mutants are longer and thicker, while there is no difference between heterozygous and wild-type mice [22]. Further research has found that the *HOXB13* gene coordinates cell death and proliferation. It is involved in regulating cell death in the tail spinal cord and also inhibits the proliferation of neuronal cells in the secondary neural tube [22]. The *HOXB13* gene is a key regulatory factor in the development of the caudal vertebrae, and its overexpression significantly reduces the rate of tail bud elongation [23]. Conversely, its inhibitory effect results in the extension of the caudal vertebrae [22,24]. Another study identified a 168 bp SINE element insertion in the upstream 5′ UTR region of the *HOXB13* gene. The 168 bp insertion variant sheep generally have longer tails than the deletion type, and this 168 bp insertion is a candidate causal variation for long tail in sheep [19]. Afterwards, in order to evaluate the impact of the 168 bp insertion on protein translation efficiency, our team conducted cell transfection experiments and found that the insertion significantly reduced the protein translation efficiency [19]. In humans, *HOXB13* is associated with the development of various cancers. The activity of the *HOXB13* transcription factor is regulated by cofactors and other transcription factors, which together regulate downstream target genes at the transcriptional level, thereby affecting the proliferation, apoptosis, migration, and invasion of tumor cells [25].

The above research suggests the impact of this gene on animals and humans. Research on this gene in human tumors is relatively comprehensive, but research in animals primarily focuses on tail length traits. The aims of this study are to evaluate the frequency of *HOXB13* gene mutations in different sheep breeds around the world and to investigate the impact of the 168 bp insertion upstream of the *HOXB13* gene on growth traits. By studying its molecular markers, a theoretical basis can be provided for sheep MAS breeding, promoting the improvement of the industry and fostering genetic enhancement and sustainable development within the breeding sector.

## 2. Materials and Methods

### 2.1. Ethics Statement

Sample collection was conducted in accordance with the Chinese national standard “Guidelines for the Welfare and Ethical Review of Experimental Animals” (GB/T 35892-2018 [26]), and the experiment was approved by the Regulations on the Management of Experimental Animals at Northwest A&F University (NWAFU-314020038).

### 2.2. Animal Sample Collection

A total of 3980 sheep were utilized in this study. Firstly, this study utilized whole-genome sequencing (WGS) data, which included 588 samples from 33 different sheep breeds worldwide, such as 54 East Friesian Dairy Sheep, 25 Tibetan Sheep, and 22 Bashibai Sheep, with each breed having a population of over 10 (*n* ≥ 10). Our team provided comprehensive details on the mode of collection of sheep samples and genotyping procedures in another study, which can be used to assess the frequency of *HOXB13* gene mutations in different sheep breeds worldwide [19]. Secondly, we selected 3392 sheep from six breeds: Guiqian semi-fine wool sheep (*n* = 590; Bijie, China), Yuansheng milk sheep (*n* = 253; Jinchang, China), Lanzhou fat-tailed sheep (*n* = 46; Lanzhou, China), Luxi black-headed sheep (*n* = 631; Liaocheng, China), Aoduhu hybrid sheep (*n* = 1123; Inner Mongolia, China), and Australian white sheep (*n* = 749; Tianjin, China). For each breed, we selected sheep that were raised on the same farm, with consistent environmental and management conditions. We collected ear tissue samples from each individual, preserved them in 70% ethanol, and kept them at low temperature in an ice box before placing them in a −80 °C freezer for storage. Among the 631 Luxi black-headed sheep, data on body size were available for 590. Of these, 33.2% (*n* = 196) were male sheep, and 66.8% (*n* = 394) were female sheep, including 100 adult females (≥1 year old). We measured and recorded various growth traits for the adult ewes of the Luxi black-headed sheep, including body weight, body height, body length, hip cross height, chest depth, chest width, chest girth, abdomen circumference, cannon (bone) circumference, and hip width. All measurements were taken accurately by the same person by using consistent methods. The measurement tools included disinfected electronic scales, vernier calipers, etc. The data were documented in an electronic spreadsheet.

### 2.3. Extraction of Genomic DNA

Genomic DNA was extracted from sheep ear tissue by using the high-salt method, and the concentration and purity of the DNA were measured. Qualified DNA was then used in subsequent experiments. The DNA sample was diluted to 20 ng/μL with distillation–distillation H_2_O (ddH_2_O).

### 2.4. Primer Design

Firstly, a 168 bp SINE element insertion upstream of the *HOXB13* gene was identified from the literature [19]. Subsequently, three pairs of primers (Table 1) were designed by using NCBI Primer-Blast (https://www.ncbi.nlm.nih.gov/tools/primer-blast/, accessed on 16 July 2023) based on the reference sequence of the sheep *HOXB13* gene (NC: 056064.1). These primers were synthesized by Sangon Biotech (Shanghai) Co., Ltd. (Shanghai, China).

### 2.5. InDel Detection and Genotyping

Polymerase chain reaction (PCR) was used to amplify polymorphic fragments, and the corresponding reaction program (pre-denaturation at 95 °C for 3 min; denaturation at 94 °C for 15 s, annealing at 60 °C for 30 s, extension at 72 °C for 30 s, 40 cycles; final extension at 72 °C for 10 min and 10 s; cooling at 12 °C for 5 min) and a PCR amplification system with a volume of 13 µL were employed. The genotypes of different individuals were identified by agarose gel electrophoresis with a 2.5% mass concentration, and the representative genotypes were sequenced by Sangon Biotech (Shanghai) Co., Ltd., to verify the mutation.

### 2.6. Whole-Genome Sequencing (WGS)

In order to study the distribution of the *HOXB13* gene in different sheep breeds worldwide, a sequencing dataset of 588 individuals from 33 sheep breeds across various geographical regions was obtained by using the whole-genome sequencing (WGS) methodology. The sample and data collection information related to the sheep population are described in another study by our team [19].

### 2.7. Statistical Analysis

Genotype frequency and allele frequency were calculated by using Excel (Version: 2019). The Genetic Diversity Index Calculator website (http://www.msrcall.com/Gdicall.aspx, accessed on 22 February 2024) was used to calculate homozygosity (Ho), heterozygosity (He), number of effective alleles (Ne), polymorphic information content (PIC), and Hardy–Weinberg equilibrium (*p*-value) [27]. Chi-square analysis was applied to analyze the differences in genotype frequency and allele frequency among different sheep breeds. By using SPSS (Version: 27) software, the association analysis between different genotypes at the InDel locus of the *HOXB13* gene and growth traits was conducted by using independent samples T-test methods.

## 3. Results

### 3.1. Analysis of HOXB13 Gene Distribution in 33 Sheep Breeds Worldwide

**Figure 1:**
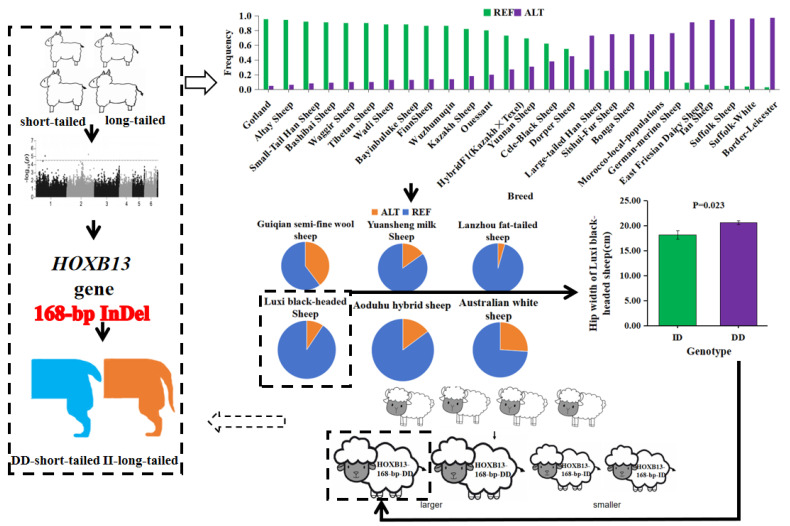
The research design and results.

The distribution of the *HOXB13* gene among 33 sheep breeds worldwide, collected based on whole-genome sequencing (WGS) data, is shown in Table 2 and Figure 1. Through association analysis and the Kruskal-Wallis H test, we analyzed whether there were differences in the frequency of the “I” allele among different regions. As can be seen from Table A1 and Table A2, the frequency of the “I” allele varies significantly across different regions. Among them, breeds from Europe and Oceania exhibited higher frequencies.

### 3.2. InDel Genotyping and Sequencing

**Figure 2:**
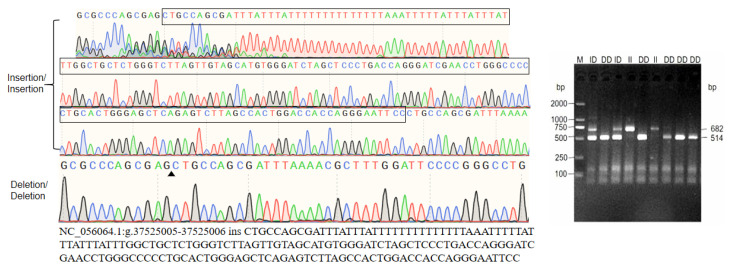
Genotyping and sequencing results.

The results of genotyping and sequencing indicate that there are three genotypes at the 168 bp inserted InDel site: II (insertion/insertion, 682 bp/682 bp), ID (insertion/deletion, 682 bp/514 bp), and DD (deletion/deletion, 514 bp/514 bp) (Figure 2).

### 3.3. Genetic Parameter Analysis

The genotype frequency, allele frequency, and population genetic indicators of the 168 bp InDel locus in the *HOXB13* gene for six sheep breeds are summarized in Table 3. The frequency of allele “D” at the 168 bp InDel locus is higher than that of allele “I” in all varieties. Among all tested sheep breeds, the 168 bp InDel locus exhibits low genetic polymorphism (PIC < 0.25) in Yuansheng milk sheep, Lanzhou fat-tailed sheep, Luxi black-headed sheep, and Aoduhu hybrid sheep, and moderate genetic polymorphism (0.25 < PIC < 0.5) in Guiqian semi-fine wool sheep and Australian white sheep. Among all the sheep breeds tested, the 168 bp InDel locus maintains Hardy–Weinberg equilibrium (*p* > 0.05).

### 3.4. Chi-Square Analysis

By applying chi-square analysis, we analyzed whether there were differences in genotype frequency and allele frequency among different sheep breeds. The analysis results show that except for the insignificant difference in genotype frequency between Lanzhou fat-tailed sheep and Luxi black-headed sheep, there were significant differences in genotype frequency among other sheep breeds. There was no significant difference in allele frequency between Lanzhou fat-tailed sheep and Yuansheng milk sheep, Luxi black-headed sheep, and Aoduhu hybrid sheep; similarly, there was no significant difference in allele frequency between Yuansheng milk sheep and Aoduhu hybrid sheep. However, there was a significant difference in allele frequency among other different sheep breeds (Table 4).

### 3.5. Association Analysis between 168 bp InDel Locus of HOXB13 Gene and Growth Traits of Luxi Black-Headed Sheep

We analyzed the relationship between the 168 bp InDel locus of the *HOXB13* gene and growth traits in Luxi black-headed sheep (Table 5; Figure 1). The independent samples T-test results show that there were no significant differences in body weight, body height, body length, hip cross height, chest depth, chest width, chest girth, abdomen circumference, and cannon (bone) circumference in adult ewes (*p* > 0.05). There was a significant difference in hip width among adult ewes (*p* < 0.05), with those having the DD genotype having significantly larger hip width than those with the ID genotype. Therefore, we believe that Luxi black-headed sheep with the DD genotype exhibit better growth conditions than those with the ID genotype.

## 4. Discussion

In our team’s recent research, we utilized GWAS to discover the *HOXB13* gene. Subsequently, through further research, we found a 168 bp SINE element insertion in the upstream 5′ UTR region of the *HOXB13* gene. We also discovered that this 168 bp insertion is associated with the sheep long-tail phenotype. This finding prompted us to consider the distribution of this 168 bp insertion among sheep breeds worldwide and whether it is related to growth traits in addition to its impact on tail length traits. This study preliminarily identified a correlation between the 168 bp InDel locus within the *HOXB13* gene and growth traits in sheep. Future research should validate the observed association between the *HOXB13* gene 168 bp InDel locus and growth traits in a broader range of samples and diverse populations and continue to explore other potential genetic markers to deepen our understanding of the genetic basis of growth traits in sheep. It is worth noting that this study reports for the first time a substantial association between the InDel polymorphism of the *HOXB13* gene and sheep growth traits.

The *Hox* gene encodes transcriptional regulatory proteins that control the axial patterns of all bilateral animals [28,29]. The *Hox* gene is believed to contribute to the anteroposterior (a-p) pattern during embryonic development in vertebrates, playing an important role in the axial development pattern of organisms [30]. Mammals have nearly 40 members of the *Hox* gene family, divided into four clusters: *HoxA*, *HoxB*, *HoxC*, and *HoxD*. The formation of these genes is mainly caused by the duplication of ancestral clusters and subsequent gene loss or duplication [31,32]. *HOXB13* is the most 5′ gene in the *HoxB* cluster, which is related to tail formation [21,22]. *HOXB13* is well expressed in the development and regeneration of the forelimbs, hindlimbs, and tail of salamanders (Mexican salamanders) [22,33]. 

In this study, we used whole-genome sequencing (WGS) data to analyze the frequency of the *HOXB13* gene in different sheep breeds. To enhance the significance of our results in terms of their use for evaluating the frequency of *HOXB13* gene variation, we selected 588 samples from 33 different sheep breeds from various regions around the world. We found that the expression of the *HOXB13* gene shows regional specificity: it is expressed more widely in sheep breeds from Europe and Oceania. This phenomenon may be caused by the different uses of sheep and differences in the selection of required traits. In addition, we genotyped six specific sheep breeds by PCR and agarose gel electrophoresis and then sequenced the representative genotypes to verify the results. We associated the polymorphism of the *HOXB13* gene with the growth traits of Luxi black-headed sheep and found a significant correlation between the *HOXB13* gene and the hip width trait in Luxi black-headed sheep.

At present, most modern sheep breeds are long-tailed, but due to a series of defects such as susceptibility to diseases and poor wool quality [2], short-tailed sheep are more inclined to be cultivated in actual production. Therefore, we believe that the 168 bp deletion genotype of the *HOXB13* gene is the dominant genotype in sheep. Hip width refers to the distance between the outer edges of the two hip angles, and it is an important growth trait in animals [34]. Hip width is not only related to reproductive performance but can also serve as an indicator for evaluating sheep’s body size and muscle development [35]. A wider hip bone means a larger pelvic cavity, which helps with childbirth and may increase fertility rates. Generally speaking, animals with wider hip bones have better meat bodies, which may be related to muscle distribution and fat deposition, thereby affecting meat yield and quality. Therefore, we believe that sheep breeds with larger hip width should be bred. This study indicates that the hip width of adult ewes with the DD genotype is significantly greater than that of adult ewes with the ID genotype, and the DD genotype is the dominant one. Our team’s previous research has shown that sheep with insertion genotypes have longer tails, while long tails have a series of defects, so we believe that the DD genotype is the dominant one for tail length traits [19]. Therefore, the research results on sheep tail length traits and growth traits are consistent. Collaborative selection could be employed in breeding, and breeding sheep with the DD genotype may lead to a better meat body shape and a shorter tail length. This study provides theoretical and experimental support for accelerating sheep breeding at the molecular level [19]. In addition, further investigations are needed to determine the specific mechanism by which the *HOXB13* gene affects growth traits. Additional research is also necessary to establish whether there is a physiological correlation between tail length traits and growth traits.

## 5. Conclusions

This study detected a functional 168 bp element insertion upstream of the *HOXB13* gene in multiple breeds of sheep. In addition, the 168 bp InDel locus is significantly correlated with the hip width trait of Luxi black-headed sheep, indicating that this InDel mutation locus could serve as a DNA marker for assisted selection in sheep in the future, which may promote breed improvements in sheep.

## Figures and Tables

**Table 1:** Primers of the sheep *HOXB13* gene used for genotyping.

Variant ID	Primer Names	Primer Sequences (5′ to 3′)	Tm (°C)	Product Sizes (bp)
INS.57332	P1	F: TTATGAGCTTCTCTCCGCCAG	59.59	514/682
		R: CTTCAGCGAGCTTCGAGACA	60.11	
	P2	F: CTGGGTTGTTCCCAACTGGA	59.82	510/678
		R: AGCCTTCAATCTCCTTGGCG	60.39	
	P3	F: CCGACGTAGCTGGGTTGTTC	61.01	488/656
		R: GGTATAATTGCCGGGCTCCAT	60.27	

**Table 2:** Genotypic frequency and allele frequency of the *HOXB13* gene 168-bp InDel locus among 33 sheep breeds worldwide.

Region	Breed	Tail Type	Sample Size	Breed Type	DD	ID	II	I	D
Wild	Mouflon	Short thin-tailed	*n* = 33	Wild sheep	1.00	0.00	0.00	0.00	1.00
Oceania	Border–Leicester	-	*n* = 19	Meat–wool	0.00	0.05	0.95	0.97	0.03
Europe	Gotland	Short thin-tailed	*n* = 10	Meat–wool	0.90	0.10	0.00	0.05	0.95
	Ouessant	Short thin-tailed	*n* = 10	Wool	0.70	0.20	0.10	0.20	0.80
	Suffolk–White	Long thin-tailed	*n* = 13	Meat–wool	0.00	0.08	0.92	0.96	0.04
	Solognote	Long thin-tailed	*n* = 10	Meat	0.00	0.00	1.00	1.00	0.00
	FinnSheep	Short thin-tailed	*n* = 11	Wool	0.73	0.27	0.00	0.14	0.86
	Suffolk Sheep	Long thin-tailed	*n* = 11	Meat	0.00	0.09	0.91	0.95	0.05
	German merino Sheep	Long thin-tailed	*n* = 21	Wool	0.10	0.29	0.62	0.76	0.24
	Poll–Dorset	Long thin-tailed	*n* = 21	Meat	0.00	0.00	1.00	1.00	0.00
	East Friesian Dairy Sheep	Long thin-tailed	*n* = 54	Milk	0.06	0.07	0.87	0.91	0.09
East Asia	Tibetan Oula Sheep	-	*n* = 10	Meat	1.00	0.00	0.00	0.00	1.00
	Waggir Sheep	Short fat-tailed	*n* = 10	Meat	0.80	0.20	0.00	0.10	0.90
	Sishui Fur Sheep	Long fat-tailed	*n* = 10	Lambskin–meat	0.20	0.10	0.70	0.75	0.25
	Wuzhumuqin	-	*n* = 11	Meat–fat	0.82	0.09	0.09	0.14	0.86
	Large-tailed Han Sheep	Long fat-tailed	*n* = 11	Meat–fat–lambskin–wool	0.00	0.55	0.45	0.73	0.27
	Small-Tail Han Sheep	Short fat-tailed	*n* = 12	Lambskin–meat	0.83	0.17	0.00	0.08	0.92
	Wadi Sheep	Short fat-tailed	*n* = 12	Meat–wool	0.75	0.25	0.00	0.13	0.88
	HybridF1(Kazakh×Texel)	-	*n* = 15	Meat	0.53	0.40	0.07	0.27	0.73
	Bayinbuluke Sheep	Fat-rumped	*n* = 16	Meat–fat	0.75	0.25	0.00	0.13	0.88
	Kazakh Sheep	Fat-rumped	*n* = 19	Meat–fat	0.68	0.26	0.05	0.18	0.82
	Tan Sheep	Long fat-tailed	*n* = 17	Wool	0.06	0.00	0.94	0.94	0.06
	Yunnan Sheep	Short thin-tailed	*n* = 21	Meat–wool–lambskin	0.48	0.43	0.10	0.31	0.69
	Duolang Sheep	Short fat-tailed	*n* = 22	Meat–fat	1.00	0.00	0.00	0.00	1.00
	Bashibai Sheep	Fat-rumped	*n* = 22	Wool–meat	0.86	0.09	0.05	0.09	0.91
	Cele Black Sheep	Short fat-tailed	*n* = 26	Lambskin	0.38	0.46	0.15	0.38	0.62
	Altay Sheep	Fat-rumped	*n* = 27	Meat	0.93	0.04	0.04	0.06	0.94
	Tibetan Sheep	Short thin-tailed	*n* = 25	Coarse wool	0.80	0.20	0.00	0.10	0.90
	Hu Sheep	Short fat-tailed	*n* = 46	Meat	1.00	0.00	0.00	0.00	1.00
Africa	Bonga Sheep	-	*n* = 10	Meat	0.00	0.50	0.50	0.75	0.25
	Morocco local populations	Long thin-tailed	*n* = 10	Meat	0.00	0.50	0.50	0.75	0.25
	Kefis Sheep	-	*n* = 13	Lambskin	1.00	0.00	0.00	0.00	1.00
	Dorper Sheep	-	*n* = 10	Meat	0.40	0.30	0.30	0.45	0.55

Note: II, insertion/insertion; ID, insertion/deletion; DD, deletion/deletion. The population size of each sheep breed is greater than or equal to 10 (*n* ≥ 10).

**Table 3:** Genetic parameters of 168 bp InDel in six different sheep types.

	Guiqian Semi-Fine Wool Sheep	Yuansheng Milk Sheep	Lanzhou Fat-Tailed Sheep	Luxi Black-Headed Sheep	Aoduhu Hybrid Sheep	Australian White Sheep
Sample Size	590	253	46	631	1123	749
II	0.207 (*n* = 122)	0.012 (*n* = 3)	0.000 (*n* = 0)	0.016 (*n* = 10)	0.038 (*n* = 43)	0.042 (*n* = 31)
ID	0.380 (*n* = 224)	0.277 (*n* = 70)	0.087 (*n* = 4)	0.155 (*n* = 98)	0.220 (*n* = 247)	0.435 (*n* = 326)
DD	0.413 (*n* = 244)	0.711 (*n* = 180)	0.913 (*n* = 42)	0.829 (*n* = 523)	0.742 (*n* = 833)	0.523 (*n* = 392)
I	0.397	0.151	0.044	0.094	0.148	0.259
D	0.603	0.849	0.956	0.906	0.852	0.741
Ho	0.521	0.744	0.916	0.830	0.747	0.616
He	0.479	0.256	0.084	0.170	0.253	0.384
Ne	1.919	1.345	1.092	1.205	1.338	1.623
PIC	0.364	0.224	0.081	0.156	0.221	0.310
HWE *p*-value	0.672	0.424	0.180	0.312	0.420	0.572

Note: II, insertion/insertion; ID, insertion/deletion; DD, deletion/deletion; Ho, homogeneity; He, heterozygosity; Ne, effective allele number; PIC, polymorphism information content; HWE, Hardy–Weinberg equilibrium.

**Table 4:** Chi-square analysis for genotype frequency and allele frequency of different sheep breeds.

Breed	Guiqian Semi-Fine Wool Sheep	Yuansheng Milk Sheep	Lanzhou Fat-Tailed Sheep	Luxi Black-Headed Sheep	Aoduhu Hybrid Sheep	Australian White Sheep
Guiqian semi-fine wool sheep		1.5634 × 10^−18^ **	3.7934 × 10^−10^ **	7.2345 × 10^−54^ **	1.1586 × 10^−47^ **	3.0586 × 10^−20^ **
Yuansheng milk sheep	4.3482 × 10^−13^ **		0.015 *	0.000173 **	0.024 *	6.1185 × 10^−7^ **
Lanzhou fat-tailed sheep	1.2154 × 10^−7^ **	0.058		0.298	0.028 *	0.000002 **
Luxi black-headed sheep	1.3407 × 10^−36^ **	0.017 *	0.419		0.000065 **	8.5599 × 10^−32^ **
Aoduhu hybrid sheep	1.5138 × 10^−29^ **	0.922	0.052	0.001 **		1.2266 × 10^−22^ **
Australian white sheep	1.0143 × 10^−7^ **	0.000289 **	0.000319 **	6.6551 × 10^−16^ **	3.7062 × 10^−9^ **	

Note: * *p* < 0.05 and ** *p* < 0.01; the section above the diagonal displays genotype frequencies, while the section below the diagonal presents allele frequencies.

**Table 5:** Association analysis between *HOXB13* 168 bp InDel locus and growth traits of Adult Luxi black-headed ewe.

Traits	Sample Size	Observed Genotypes (Mean ± SE)		*p*-Value
		ID	DD	
Hip width (cm)	98	18.17 ± 0.84	20.64 ± 0.38	0.023
Body weight (kg)	100	60.67 ± 4.62	64.48 ± 1.44	0.373
Body height (cm)	100	67.25 ± 1.18	68.72 ± 0.42	0.233
Body length (cm)	100	75.58 ± 1.56	76.35 ± 0.63	0.670
Hip cross height (cm)	100	69.83 ± 1.10	69.94 ± 0.38	0.920
Chest depth (cm)	100	30.30 ± 0.95	33.32 ± 2.70	0.682
Chest width (cm)	100	21.64 ± 1.44	23.96 ± 0.51	0.118
Chest girth (cm)	100	96.42 ± 3.10	96.59 ± 0.98	0.952
Abdomen circumference (cm)	100	117.92 ± 2.67	118.32 ± 0.96	0.885
Cannon (bone) circumference (cm)	100	9.08 ± 0.33	9.26 ± 0.10	0.561

Note: The *p*-value represents the probability that the differences between samples are caused by sampling error.

## Data Availability

The data presented in this study are available in the article.

## Appendix A

**Table A1:** Association analysis between different regions and allele frequency of “I” in sheep.

Trait	Sample Size	Regions (Mean ± SE)					*p*-Value
		Wild	Oceania	Europe	East Asia	Africa	
Frequency	33	0.00 ± 0.00 (*n* = 1)	0.97 ± 0.00 (*n* = 1)	0.66 ± 0.14 (*n* = 9)	0.24 ± 0.07 (*n* = 18)	0.49 ± 0.18 (*n* = 4)	0.017

**Table A2:** The results of the Kruskal-Wallis H test for the frequency of the "I" allele in sheep breeds from different regions.

Null Hypothesis	Test	Significance	Decision
The distribution of I is the same across categories of regions	Independent samples Kruskal-Wallis test	0.023	Reject the null hypothesis
